# Decision making in treatment of symptomatic severe aortic stenosis: a survey study in Dutch heart centres

**DOI:** 10.1007/s12471-022-01676-w

**Published:** 2022-04-05

**Authors:** J. J. A. M. van Beek-Peeters, Z. van den Ende, M. C. Faes, A. J. B. M. de Vos, M. W. A. van Geldorp, B. J. L. Van den Branden, B. J. M. van der Meer, M. M. N. Minkman

**Affiliations:** 1 Department of Cardiothoracic Surgery, Amphia Hospital, Breda, The Netherlands; 2 Department of Geriatrics, Amphia Hospital, Breda, The Netherlands; 3 Amphia Academy, Amphia Hospital, Breda, The Netherlands; 4 Department of Interventional Cardiology, Amphia Hospital, Breda, The Netherlands; 5 TIAS School for Business and Society, Tilburg University, Tilburg, The Netherlands; 6 Board of Directors, Catharina Hospital, Eindhoven, The Netherlands; 7 Vilans, Centre of Expertise for Long-term Care, Utrecht, The Netherlands

**Keywords:** Heart team, Aortic valve replacement, Decision making, Frailty

## Abstract

**Aim:**

To provide insight into the basic characteristics of decision making in the treatment of symptomatic severe aortic stenosis (SSAS) in Dutch heart centres with specific emphasis on the evaluation of frailty, cognition, nutritional status and physical functioning/functionality in (instrumental) activities of daily living [(I)ADL].

**Methods:**

A questionnaire was used that is based on the European and American guidelines for SSAS treatment. The survey was administered to physicians and non-physicians in Dutch heart centres involved in the decision-making pathway for SSAS treatment.

**Results:**

All 16 Dutch heart centres participated. Before a patient case is discussed by the heart team, heart centres rarely request data from the referring hospital regarding patients’ functionality (*n* = 5), frailty scores (*n* = 0) and geriatric consultation (*n* = 1) as a standard procedure. Most heart centres ‘often to always’ do their own screening for frailty (*n* = 10), cognition/mood (*n* = 9), nutritional status (*n* = 10) and physical functioning/functionality in (I)ADL (*n* = 10). During heart team meetings data are ‘sometimes to regularly’ available regarding frailty (*n* = 5), cognition/mood (*n* = 11), nutritional status (*n* = 8) and physical functioning/functionality in (I)ADL (*n* = 10). After assessment in the outpatient clinic patient cases are re-discussed ‘sometimes to regularly’ in heart team meetings (*n* = 10).

**Conclusions:**

Dutch heart centres make an effort to evaluate frailty, cognition, nutritional status and physical functioning/functionality in (I)ADL for decision making regarding SSAS treatment. However, these patient data are not routinely requested from the referring hospital and are not always available for heart team meetings. Incorporation of these important data in a structured manner early in the decision-making process may provide additional useful information for decision making in the heart team meeting.

**Supplementary Information:**

The online version of this article (10.1007/s12471-022-01676-w) contains supplementary material, which is available to authorized users.

## What’s new?

- Heart centres do not routinely request data regarding frailty, cognition, nutritional status and physical functioning/functionality in (instrumental) activities of daily living ((I)ADL) from the referring hospital, but most heart centres do their own screening for these factors.
- Data regarding frailty, cognition, nutritional status and physical functioning/functionality in (I)ADL are not always available for heart team meetings.
- Incorporating these data in a structured manner early in the decision-making process may add information that is useful for decision making in the heart team meeting.

## Introduction

Decision making in the treatment of symptomatic severe aortic valve stenosis (SSAS) is challenging. Guidelines for SSAS recommend a thorough evaluation of essential patient-related factors: symptoms and severity of SSAS, comorbidity, life expectancy, quality of life, treatment options as well as the benefits of these options and patient preferences [1–4]. In addition, there is growing interest in the assessment of frailty, cognition, nutritional status and functionality, which facilitates the identification of patients at high risk of complications from surgical aortic valve replacement (SAVR) and transcatheter aortic valve replacement (TAVR) and guides peri-operative optimisation strategies [1, 4, 5].

Frailty is a condition with a high prevalence in older persons and is characterised by a decline in multiple physiological systems and increased vulnerability to stressors [6]. Frailty is related to adverse health outcomes, such as falls, functional decline, hospital admissions, and is associated with increased morbidity and mortality after TAVR and SAVR [7, 8]. Functionality in daily living is the ability to perform self-care activities of daily living (ADL). ADL tasks have been classified into (1) basic activities of daily living (basic ADL) that a person normally performs on a daily basis, such as walking and (2) instrumental activities of daily living (IADL) that allow an individual to live independently in a community, such as doing grocery shopping [9]. Physical functioning is described as the physical capacities of patients, for example walking distance, walking speed or hand grip strength [10]. Several screening instruments for assessing frailty, cognition, nutritional status, physical functioning and functionality are available [1, 11].

To facilitate the decision-making process, European and American guidelines recommend a multidisciplinary heart team approach [1, 3, 4, 12, 13]. This dedicated heart team optimises patient selection for either conservative treatment (CT), TAVR or SAVR, through a comprehensive understanding of the risk-benefit ratio of different options, thereby taking into account the patient’s values and preferences [3, 4, 13]. The core of this heart team for SSAS treatment preferably consists of a cardiothoracic surgeon and an interventional cardiologist. However, a more extensive multidisciplinary team is preferred in complex cases [3, 4]. Additionally, the expert consensus decision (ECD) pathway for TAVR divides all aspects of decision making into essential key steps in patient selection and evaluation (e.g. approach to care, goals of care, initial assessment and functional assessment) [13].

Nevertheless, decision making regarding SSAS treatment remains complex due to multiple treatment options, patient characteristics and personal preferences of both patients and health care professionals [14, 15]. It is unclear which health care professionals participate in decision making, which data drive the decision-making process and when these data are obtained [12]. More specifically, little is known about the use of screening instruments for frailty, cognition, nutrition and physical functioning/functionality in (I)ADL in decision making. Therefore, this retrospective descriptive study provides insight into the basic characteristics of the decision-making structure of SSAS treatment in Dutch heart centres with specific emphasis on the evaluation of frailty, cognition, nutritional status and physical functioning/functionality in (I)ADL. Additionally, differences and similarities between Dutch heart centres are studied.

## Methods

### Design

For this descriptive study, a retrospective cross-sectional design was used. An online survey was administered to physicians and nurse practitioners/physician assistants in Dutch heart centres involved in the decision-making pathway regarding patients referred for treatment of SSAS.

### Instrument

A questionnaire was used that is based on the European and American guidelines for SSAS treatment. An expert panel comprising a cardiothoracic surgeon, a geriatrician, a cardiologist and two senior researchers reviewed and piloted the tailored self-administered questionnaire for relevance to the basic characteristics regarding the decision-making structure of SSAS treatment and the evaluation of frailty, cognition, nutritional status and physical functioning/functionality in (I)ADL [1–4].

After reviewing and piloting, four questions were added to better reflect the aim of the study. The 42 questions included 6 about demographics and 36 divided into four sections according to the key steps of patient selection and evaluation in the ECD pathway for TAVR [13]: (1) data provided by the referring cardiologist (5 items); (2) the decision-making structure and the professionals involved (7 items); (3) guidelines and care path (16 items); (4) screening of frailty, cognition and mood, nutritional status, physical functioning/functionality in ADL and IADL in the referring hospital and the heart centre (8 items). The questionnaire consisted of single-choice and multiple-choice questions, 6‑point Likert scales (range from ‘never’ to ‘always’) and open questions. For a detailed description of the questionnaire, see the Electronic Supplementary Material.

### Participants and setting

In the Netherlands 16 heart centres combine heart surgery and interventional cardiology, 8 academic and 8 large teaching hospitals. To study the full landscape, we contacted all 16 heart centres. Both physicians and nurse practitioners/physician assistants involved in the decision-making pathway were invited to participate in the study.

### Data collection/procedure

After giving their informed consent the participants received the online questionnaire and instructions. A reminder was sent after 2 weeks. Data collection took place from June until September 2019.

### Statistical analysis

Quantitative data were analysed using descriptive statistics, with IBM-SPSS 26 (IBM Corp., Armonk, NY, USA). Discrete variables are presented as counts and percentages. The 6‑point Likert scales were merged into three categories (seldom to never, sometimes to regularly and often to always). Qualitative data were analysed through thematic analysis, to identify categories and themes [16].

A comparative sub-analysis was executed to analyse differences between academic and large teaching hospitals as regards care structure and context.

## Results

All 16 heart centres participated in the study. Twelve physicians and four nurse practitioners/physician assistants completed the questionnaire (Tab. 1).

**Table 1:** Characteristics of respondents

	Academic hospitals (*n* = 8)	Large teaching hospitals (*n* = 8)	Total (*n* = 16)
	*n* (%)	*n* (%)	*n* (%)
Profession			
Cardiothoracic surgeon	2 (25)	2 (25)	4 (25)
Interventional cardiologist	3 (38)	4 (50)	7 (44)
Nurse practitioner	1 (13)	2 (25)	3 (19)
Physician assistant	1 (13)	0 (0)	1 (6)
Cardiothoracic surgeon in training	1 (13)	0 (0)	1 (6)

### Structures and professionals involved

In all heart centres (*n* = 16) the interventional cardiologist and cardiothoracic surgeon always participate in the heart team meetings (Electronic Supplementary Material, Table S1). In two heart centres a geriatrician participates. More than half of the heart centres (*n* = 11) have a multidisciplinary team for TAVR in addition to the heart team; three heart centres have an additional multidisciplinary team for SAVR (Electronic Supplementary Material, Table S1).

### Guidelines and care paths

The guidelines most often used for decisions regarding SSAS treatment are: the 2017 guidelines of the European Society of Cardiology/European Association for Cardio-Thoracic Surgery [1] (*n* = 14), the ‘Indications for TAVR’ document of the Netherlands Society of Cardiology/Netherlands Society of Thoracic Surgery [17] (*n* = 8) and the ‘Moments of decision’ paper of the Netherlands Society of Thoracic Surgery [2] (*n* = 5) (Electronic Supplementary Material, Table S2).

Ten heart centres use a care path for both SAVR and TAVR and six heart centres only for TAVR (Electronic Supplementary Material, Table S1).

The most common pre-operative model to assess patients for both TAVR and SAVR is the outpatient clinic with a carousel approach (*n* = 9) (Electronic Supplementary Material, Table S2), where professionals assess the patient consecutively during one patient visit. In nine heart centres the cardiothoracic surgeon participates in a carousel outpatient clinic and the interventional cardiologist participates at four heart centres (Electronic Supplementary Material, Table S2). Most professionals assess patients in the outpatient clinic after the heart team meeting (Electronic Supplementary Material, Table S2).

The most common reasons for postponing the treatment decision sometimes to regularly include: additional examinations required (*n* = 15), consultations other than with cardiology (*n* = 15) and vitality issues (*n* = 14) (Electronic Supplementary Material, Table S2). After assessment in the outpatient clinic, patient cases are re-discussed sometimes to regularly in heart team meetings (*n* = 10) and recommending conservative treatment occurs never to seldom in more than half of the heart centres (*n* = 11). After the heart team has determined the indication for treatment, the first treatment advice is never to seldom changed in ten heart centres (Tab. 2).

**Table 2:** Evaluation of treatment after assessment in outpatient clinic and after first treatment advice

	Academic hospitals (*n* = 8)	Large teaching hospitals (*n* = 8)	Total (*n* = 16)
	*n* (%)	*n* (%)	*n* (%)
*Re-discussion in heart team after assessment in outpatient clinic*			
Seldom to never	4 (50)	1 (13)	5 (31)
Sometimes to regularly	4 (50)	6 (75)	10 (63)
Often to always	0 (0)	1 (13)	1 (6)
*Conservative treatment after assessment in outpatient clinic*			
Seldom to never	7 (88)	4 (50)	11 (69)
Sometimes to regularly	1 (13)	4 (50)	5 (31)
Often to always	0 (0)	0 (0)	0 (0)
*Change in recommended treatment after first treatment advice*			
Seldom to never	6 (75)	4 (50)	10 (63)
Sometimes to regularly	2 (25)	4 (50)	6 (38)
Often to always	0 (0)	0 (0)	0 (0)

### Evaluation of frailty, cognition, nutritional status and physical functioning/functionality in (I)ADL

Prior to each heart team case discussion none of the heart centres requests frailty scores as a standard procedure. One heart centre requests a consultation with a geriatrician, and five heart centres request data regarding functionality (*n* = 5) as a standard procedure (Electronic Supplementary Material, Table S1). Supplementary consultations are usually performed in the referring hospital (*n* = 13) (Electronic Supplementary Material, Table S1). In addition to the standard information for referral, one heart centre always requests data regarding frailty, cognition/mood, nutritional status and physical functioning/functionality in (I)ADL from the referring hospital (Electronic Supplementary Material, Table S3).

Most heart centres (often to always) do their own screening for frailty (*n* = 10), cognition/mood (*n* = 9), nutritional status (*n* = 10) and physical functioning/functionality in (I)ADL (*n* = 10). During heart team meetings data are sometimes to regularly present regarding frailty (*n* = 5), cognition/mood (*n* = 11), nutritional status (*n* = 8) and physical functioning/functionality in (I)ADL (*n* = 10) (Electronic Supplementary Material, Table S3).

The most frequently used screening instruments are: for frailty, the Edmonton Frail Scale (EFS) (*n* = 8); for cognition, the Mini Mental State Examination (*n* = 5); for nutritional status, the Body Mass Index (*n* = 12); and for functionality in ADL, the Katz Activities of Daily Living Scale or Barthel Index (*n* = 9) (Tab. 3).

**Table 3:** Screening instruments

Characteristics	Academic hospitals (*n* = 8)	Large teaching hospitals (*n* = 8)	Total (*n* = 16)
	*n* (%)	*n* (%)	*n* (%)
*Frailty*			
Rockwood Clinical Frailty Scale	1 (13)	0 (0)	1 (6)
Edmonton Frail Scale	2 (25)	6 (75)	8 (50)
Cardiovascular Frailty Scale	1 (13)	1 (13)	2 (13)
None	3 (38)	1 (13)	4 (25)
Other (various)	5 (63)	2 (25)	7 (44)
*Cognition or mood*			
MMSE	4 (50)	1 (13)	5 (31)
MOCA	1 (13)	0 (0)	1 (6)
GDS	2 (25)	0 (0)	2 (13)
None	2 (25)	2 (25)	4 (25)
Other (various)	3 (38)	5 (63)	8 (50)
*Nutritional status*			
Albumin	0 (0)	2 (25)	2 (13)
BMI	7 (88)	5 (63)	12 (75)
Weight last year	4 (50)	3 (38)	7 (44)
MNA	3 (38)	1 (13)	4 (25)
None	1 (13)	2 (25)	3 (19)
Other (various)	1 (13)	0 (0)	1 (6)
*Physical functioning or functionality*			
*In (instrumental) activities of daily living*			
Walking speed	2 (25)	2 (25)	4 (25)
TUG	2 (25)	1 (13)	3 (19)
Grip strength	1 (13)	1 (13)	2 (13)
Katz Activities of Daily Living Scale or Barthel Index	5 (63)	4 (50)	9 (56)
Lawton Instrumental Activities of Daily Living Scale	2 (25)	1 (13)	3 (19)
None	2 (25)	2 (25)	4 (25)
Other (various)	1 (13)	1 (13)	2 (13)

*MMSE* Mini-Mental State Examination, *MOCA* Montreal Cognitive Assessment, *GDS* Geriatric Depression Scale, *BMI* body mass index, *MNA* Mini Nutritional Assessment, *TUG* Timed Up and Go test

### Differences between academic and large teaching hospitals

In academic hospitals a diversity of screening instruments for frailty are used, while six large teaching hospitals use the EFS score (Tab. 3).

## Discussion

This study provides insight into the basic characteristics regarding the structure of decision making in the treatment of SSAS in Dutch heart centres with specific emphasis on the evaluation of frailty, cognition, nutritional status and physical functioning/functionality in (I)ADL.

Our study demonstrates that in the majority of the heart centres patient cases are regularly re-discussed in heart team meetings (after assessment in the outpatient clinic) to clarify vitality issues. However, the first treatment advice (SAVR or TAVR) of the heart team is often followed in most heart centres.

Further, data regarding frailty, cognition, nutritional status and physical functioning/functionality in (I)ADL are not routinely requested from referring hospitals, but most heart centres do their own screening for these factors. However, these data are not always available during the heart team meetings. Further, in most large teaching hospitals the EFS score is used for frailty screening, while a diversity of screening instruments are used in academic hospitals.

### Comparison with previous studies

Our findings demonstrate that data regarding frailty, cognition, nutritional status and physical functioning/functionality in (I)ADL are not routinely requested from referring hospitals. However, it is known that frailty screening is not often performed in clinical practice, although the degree of frailty is important for defining a patient’s ability to recover after TAVR or SAVR [18–20]. Nevertheless, we found that screening often takes place after the heart team has made its treatment recommendation, which is in line with optimising pre-operative strategies [5, 21].

The absence of data from screening instruments prior to referral for SSAS treatment leads the heart centres to use an outpatient clinic for screening patients themselves. In addition, screening in the outpatient or inpatient clinic before SAVR or TAVR is strongly advised and should not replace patient visits [21]. However, data from screening instruments from referring hospitals can add useful information for identification of high-risk patients by the heart team [5].

The diversity of the frailty instruments used in academic hospitals illustrates their focus on frailty research [22]. On the other hand, limited synergy between hospitals may lead to diversity in frailty screening and may result in differences in reported frailty or complexity when patients are transferred [23, 24].

### Strength and limitations

To our knowledge, this is the first study that provides insight into the basic structures and evaluation of frailty, cognition, nutrition and physical functioning/functionality in (I)ADL for decision making regarding SSAS treatment in Dutch heart centres. The strength of this study is the participation of all Dutch heart centres, therefore providing an overview of the Dutch landscape. Nevertheless, the results should be interpreted in the light of some limitations.

First, this study relied on the answers of one medical coordinator per centre and self-reporting, which may have led to social desirability [25]. In order to mitigate social desirability, we informed respondents about the anonymous processing of the data. Subsequently, different questions for corresponding items were included in the questionnaire. Second, this study reflects the situation between June 2021 and September 2019. Current practice has changed as a result of the recent ZiN (Zorginstituut Nederland) directive, the subsequent TAVR indications guidelines and the new multidisciplinary heart team format for the treatment of SSAS [26, 27]. However, our study demonstrates the difficulties of collecting data regarding frailty, cognition, nutrition and physical functioning/functionality in (I)ADL in daily practice and may provide information for practical adjustments.

### Implications for clinical practice and future research

A first step is the need for professionals to request data regarding frailty, cognition, nutrition and physical functioning/functionality in (I)ADL in a standard format from referring hospitals and to make the data available during heart team meetings. Therefore, guidelines have to clarify standardised and valid screening instruments. Special attention is needed regarding how professionals can incorporate screening instruments for cognition, nutrition and physical functioning/functionality in (I)ADL in their daily practice and decision-making structures [20]. A geriatric consultation and a comprehensive geriatric assessment for pre-operative evaluation of patients above 75 years is recommended when a thorough assessment of frailty and functionality is needed [28, 29].

Given the high number of patients with valve disease such as SSAS, future research needs to clarify how the current situation has changed following the new ZiN directive [26, 30]. Implementation research needs to focus on the status of the incorporation of screening instruments at referring hospitals.

## Conclusion

Dutch heart centres make an effort to screen for frailty, cognition, nutritional status and physical functioning/functionality in (I)ADL for decision making regarding SSAS treatment. However, these patient data are not routinely requested from the referring hospital and are not always available for heart team meetings. Incorporation of these important data in a structured manner early in the decision-making process may add information that is useful for decision making in the heart team meeting.

## Supplementary Information

Questionnaire

Table S1. Data provision, decision-making structure and professionals involved

Table S2. Guidelines and care-paths

Table S3. Screening data in referring hospital, heart centre and heart team meeting
