# Supplementary material for: Decision making in treatment of symptomatic severe aortic stenosis: a survey study in Dutch heart centres
Source: Neth Heart J. 2022 Apr 5;30(9):423–8. doi: 10.1007/s12471-022-01676-w (PMC9402830; doi:10.1007/s12471-022-01676-w)
Supplement: Supplementary file 1 — Questionnaire [file 12471_2022_1676_MOESM1_ESM.docx]

**Electronic Supplementary Material**

**Questionnaire**

**Demographics**

Terms and abbreviations in this section:

AoS Severe aortic valve stenosis with treatment indication according to the guidelines

SAVR Surgical Aortic Valve Replacement

TAVR Transcatheter Aortic Valve Replacement (apical and femoral approach)

1. In which hospital do you work?
2. What is your profession?

- Physician in training
- Cardiothoracic surgeon
- Cardiologist
- Interventional cardiologist
- Physician assistant
- Nurse practitioner
- Nurse
- Other, e.g.

1. How many patients with AoS were discussed in the heart team in your heart centre in 2018?

- Number: ..
- Not known, but data can be requested from our datamanager: ..
- This is not reported
- These data are for internal use only

1. How many patients with AoS were treated with TAVR in your heart centre in 2018?

- Number: ..
- Not known, but data can be requested from our datamanager: ..
- This is not reported
- These data are for internal use only

1. How many patients with AoS were treated with SAVR in your centre in 2018?

- Number: ..
- Not known, but data can be requested from our datamanager: ..
- This is not reported
- These data are for internal use only

1. How many patients with AoS were advised medical treatment in your centre in 2018?

- Number: ..
- Not known, but data can be requested from our datamanager: ..
- This is not reported
- These data are for internal use only

**Data provided by the referring cardiologist**

Terms and abbreviations in this section:

AoS Severe aortic valve stenosis with treatment indication according to the guidelines

SAVR Surgical Aortic Valve Replacement

TAVR Transcatheter Aortic Valve Replacement (apical and femoral)

Care path The used decision model to standardise the routing for patients with severe aortic valve stenosis.

Heart team Indication setting team in the heart centre

1. Is a care path for aortic valve stenosis available in your heart centre?

- Yes, for TAVR
- Yes, for SAVR
- Yes, for both TAVR and SAVR
- No

1. Is an information format for the referring hospital available in your heart centre?

- Yes
- No

1. Which data are always requested at the referring hospital?

- History taking
- Physical examination
- Laboratory results
- Electrocardiogram
- Coronary angiography
- Transthoracic echocardiogram
- Frailty score
- Functionality
- Geriatric consultation
- Other, e.g.

1. Who assesses the information from the referring hospital for completeness and content prior to the heart team meeting?
2. In which hospital or centre are supplementary consultations (other than cardiology) effected?

- Referring hospital
- Heart centre

**The structure of decision-making and involved professionals**

Terms and abbreviations in this section:

Care path The used decision model to standardise the routing for patients with severe aortic valve stenosis.

Heart team Indication setting team in the heart centre

1. Which professionals are always present at heart team meetings?

- Cardiothoracic surgeon
- Interventional cardiologist
- Cardiologist
- Nurse practitioner
- Physician assistant
- Nurse
- Clinical geriatrician
- Anaesthesiologist
- Radiologist
- Other, e.g.

1. Number of professionals present at heart team meetings

*Please note a numerical value*

1. Can you describe other heart team meetings besides the heart team as indication setting team?

- Yes, e.g.
- No

1. How many times a week does the heart team meet?

*Please note a numerical value*

1. How often do consulted professionals participate in heart team meetings?

Never Seldom Sometimes Regularly Often Always

1. From how many referring hospitals patients with AoS are being discussed in the heart team?

*Please note a numerical value*

1. What is the mode of contact with the referring hospital and how often does this take place?

- Medical letter

Never Seldom Sometimes Regularly Often Always

- Digital

Never Seldom Sometimes Regularly Often Always

- Telephone

Never Seldom Sometimes Regularly Often Always

- Meeting at referring hospital

Never Seldom Sometimes Regularly Often Always

**Guidelines and care path**

Terms and abbreviations in this section:

AoS Severe aortic valve stenosis with treatment indication according to the guidelines

SAVR Surgical Aortic Valve Replacement

TAVR Transcatheter Aortic Valve Replacement (apical and femoral)

Care path The used decision model to standardise the routing for patients with severe aortic valve stenosis.

Heart team Indication setting team in the heart centre

Preoperative

Carousel Outpatient clinic where professionals assess the patient subsequently during one patient visit.

1. Which guidelines are used for decision-making regarding treatment of AoS?

*Multiple answers possible*

- Guideline for the Management of Patients With Valvular Heart Disease, AHA/ACC (American Heart Association/ American College of Cardiology)
- Guidelines for the management of valvular heart disease, ESC/EACTS (European Society of Cardiology/ European Association for Cardio-Thoracic Surgery)
- Expert Consensus Decision Pathway for Transcatheter Aortic Valve Replacement in the Management of Adults With Aortic Stenosis, ACC (American College of Cardiology)
- Moments of decision-making in the pre-, per- and postoperative trajectory of the cardiac surgical patient [Beslismomenten pre-, per- en postoperatieve traject van de hartchirurgische patiënt], Nederlandse Vereniging voor Thoraxchirurgie (NVT)
- Indicatiedocument Transcatheter Aortaklep Interventie, Nederlandse Vereniging voor Thoraxchirurgie (NVT)
- Local protocol
- Other, e.g.

1. Do you use an outpatient clinic with carousel principle in which professionals assess the patient subsequently during one patient visit?

- Yes, for SAVR
- Yes, for TAVR
- Yes, for both SAVR and TAVR
- No

1. At which moment during the preparation for treatment of AoS is the patient assessed by the following professionals?

- Cardiothoracic surgeon

Before heart team meeting After heart team meeting Carousel clinic Day before operation Not

- Interventional cardiologist

Before heart team meeting After heart team meeting Carousel clinic Day before operation Not

- Nurse practitioner/ Physician assistant

Before heart team meeting After heart team meeting Carousel clinic Day before operation Not

- Nurse

Before heart team meeting After heart team meeting Carousel clinic Day before operation Not

- Geriatrician

Before heart team meeting After heart team meeting Carousel clinic Day before operation Not

- Anaesthesiologist

Before heart team meeting After heart team meeting Carousel clinic Day before operation Not

1. Is another professional (not mentioned above) involved in the preoperative assessment of the patient?

- Yes, e.g.
- No

1. At which moment does the professional mentioned in question 21 have contact with the patient?

- E.g. ..
- Not applicable

1. Are other multidisciplinary team meetings additional to the heart team available for TAVR?

- Yes, e.g.
- No

1. Are other multidisciplinary team meetings additional to the heart team available for SAVR?

- Yes, e.g.
- No

1. How often are patients re-discussed in the heart team?

Never Seldom Sometimes Regularly Often Always

27.When the heart team cannot provide a treatment indication, patients are sometimes re-discussed at another moment. How often do the following reasons lead to re-discussion of patients?

- Consultation other than cardiology required

Never Seldom Sometimes Regularly Often Always

- Supplementary examinations required (no consultations)

Never Seldom Sometimes Regularly Often Always

- Doubts about patient’s vitality

Never Seldom Sometimes Regularly Often Always

- Patient’s symptoms not clear

Never Seldom Sometimes Regularly Often Always

28. Do other reasons lead to re-discussion of patients?

- Yes, e.g.
- No

29. How often are patients re-discussed in the heart team after assessment in the outpatient clinic?

Never Seldom Sometimes Regularly Often Always

30. How often is treatment with TAVR or SAVR changed to conservative treatment after assessment in the outpatient clinic?

Never Seldom Sometimes Regularly Often Always

31. How often are treatment proposals changed after first treatment advice in the heart team?

Never Seldom Sometimes Regularly Often Always

32. Which professional assesses the postoperative TAVR outcomes after hospital discharge?

- Interventional cardiologist
- Nurse practitioner/Physician assistant
- Referring cardiologist
- General practitioner

33. Which professional assesses the postoperative SAVR outcomes after hospital discharge?

- Cardiothoracic surgeon
- Nurse practitioner/Physician assistant
- Referring cardiologist
- General practitioner

34. What are the differences in care following hospital discharge after TAVR or SAVR?

**Screening of frailty, cognition, mood, nutritional status, physical functioning, functionality in activities of daily living and functionality in instrumental activities of daily living in referring hospital and the heart centre**

Terms and abbreviations in this section:

Frailty a state of increased vulnerability to poor resolution of homeostasis following a stress, which increases the risk of adverse outcomes including falls, delirium and disability

Heart team Indication setting team in the heart centre

ADL Activities of daily living (e.g., showering, dressing)

IADL Instrumental activities of daily living (e.g., computer, telephone)

35. Which screening instrument is used for frailty?

*Multiple answers possible*

- Rockwood clinical frailty scale
- Edmonton Frail Scale
- Cardiovascular Frailty Scale
- None
- Other, e.g.

36. Which screening instruments are used for cognition or mood?

*Multiple answers possible*

- Mini Mental State Examination
- Montreal Cognitive Assessment
- Geriatric Depression Scale
- None
- Other, e.g.

37. Which screening instrument is used for nutritional status?

*Multiple answers possible*

- Albumin
- Body Mass Index
- Weight last year
- Minimal Nutritional Assessment
- None
- Other, e.g.

38. Which screening instruments are used for physical functioning, functionality in activities of daily living (ADL) or functionality in instrumental activities of daily living (iADL)?

*Multiple answers possible*

- Walking Speed
- Timed Up and Go test
- Grip strength
- Katz activities of daily living scale/ Barthel Index
- Lawton instrumental activities of daily living scale
- None
- Other, e.g.

39. Does your heart centre request data regarding frailty, cognition/mood, nutritional status and physical functioning/functionality in daily living at the referring hospital?

*Multiple answers possible*

- Frailty

Never Seldom Sometimes Regularly Often Always

- Cognition/mood

Never Seldom Sometimes Regularly Often Always

- Nutritional status

Never Seldom Sometimes Regularly Often Always

- Physical functioning/functionality in daily living

Never Seldom Sometimes Regularly Often Always

40. Are data regarding frailty, cognition/mood, nutritional status and physical functioning/functionality in daily living assessed in your heart centre?

- Frailty

Never Seldom Sometimes Regularly Often Always

- Cognition/mood

Never Seldom Sometimes Regularly Often Always

- Nutritional status

Never Seldom Sometimes Regularly Often Always

- Physical functioning/functionality in daily living

Never Seldom Sometimes Regularly Often Always

41. Which data regarding frailty, cognition/mood, nutritional status and physical functioning/functionality in daily living are available during heart team meetings?

- Frailty

Never Seldom Sometimes Regularly Often Always

- Cognition/mood

Never Seldom Sometimes Regularly Often Always

- Nutritional status

Never Seldom Sometimes Regularly Often Always

- Physical functioning/functionality in daily living

Never Seldom Sometimes Regularly Often Always

42. Is a screening program for vulnerable patients with AoS who are being assessed for treatment available in your heart centre? (e.g., risk screening or prehabilitation program)

- Yes
- No
