# Supplementary material for: Decision making in treatment of symptomatic severe aortic stenosis: a survey study in Dutch heart centres
Source: Neth Heart J. 2022 Apr 5;30(9):423–8. doi: 10.1007/s12471-022-01676-w (PMC9402830; doi:10.1007/s12471-022-01676-w)
Supplement: Supplementary file 2 — Table S1. Data provision, decision-making structure and professionals involved [file 12471_2022_1676_MOESM2_ESM.docx]

|  | **Academic hospitals (*n*=8)** | **Large teaching hospitals (*n*=8)** | **Total (*n*=16)** |
| --- | --- | --- | --- |
|  | ***n* (%)** | ***n* (%)** | ***n* (%)** |
| **Data provision** |  |  |  |
| *Format information* |  |  |  |
| Format information for referral available | 7 (88) | 8 (100) | 15 (94) |
| *Supplementary consultations* |  |  |  |
| At referring hospital | 8 (100) | 5 (63) | 13 (81) |
| At heart centre | 0 (0) | 3 (38) | 3 (19) |
| *Available care path* |  |  |  |
| For TAVR | 3 (38) | 3 (38) | 6 (38) |
| For SAVR | 0 (0) | 0 (0) | 0 (0) |
| For both SAVR and TAVR | 5 (63) | 5 (63) | 10 (63) |
| *Data always requested at referring hospital* |  |  |  |
| Anamnesis | 8 (100) | 8 (100) | 16 (100) |
| Physical examination | 7 (88) | 7 (88) | 14 (88) |
| Laboratory results | 8 (100) | 8 (100) | 16 (100) |
| ECG | 8 (100) | 8 (100) | 16 (100) |
| CAG | 6 (75) | 8 (100) | 14 (88) |
| TTE | 8 (100) | 8 (100) | 16 (100) |
| Frailty score | 0 (0) | 0 (0) | 0 (0) |
| Functionality | 2 (25) | 3 (38) | 5 (31) |
| Geriatric consultation | 1 (13) | 0 (0) | 1 (6) |
| Lung function test | 0 (0) | 1 (13) | 1 (6) |
| **Decision-making structure** |  |  |  |
| *Additional team available* |  |  |  |
| for TAVR | 6 (75) | 5 (63) | 11 (69) |
| for SAVR | 2 (25) | 1 (13) | 3 (19) |
| *Number of referring hospitals* |  |  |  |
| 3 - 5 | 4 (50) | 2 (25) | 6 (38) |
| 6 - 8 | 2 (25) | 3 (38) | 5 (31) |
| >9 | 2 (25) | 3 (38) | 5 (31) |
| **Professionals involved** |  |  |  |
| *Professionals always present at heart team meeting* |  |  |  |
| Cardiothoracic surgeon | 8 (100) | 8 (100) | 16 (100) |
| Interventional cardiologist | 8 (100) | 8 (100) | 16 (100) |
| Cardiologist | 3 (38) | 3 (38) | 6 (38) |
| Nurse practitioner | 0 (0) | 1 (13) | 1 (6) |
| Physician assistant | 1 (13) | 0 (0) | 1 (6) |
| Nurse | 0 (0) | 0 (0) | 0 (0) |
| Clinical geriatrician | 1 (13) | 1 (13) | 2 (13) |
| Anaesthesiologist | 0 (0) | 1 (13) | 1 (6) |
| Radiologist | 0 (0) | 0 (0) | 0 (0) |
| Echocardiologist | 3 (38) | 0 (0) | 3 (19) |
| Physician in training | 0 (0) | 1 (13) | 1 (6) |
| Consulted professionals | 1 (13) | 0 (0) | 1 (6) |

**Table S1** Data provision, decision-making structure and professionals involved

*^CAG^* ^coronary angiography,^ *^ECG^* ^electrocardiogram^*^, TAVR^* ^transcatheter aortic valve replacement^*^, TTE^* ^transthoracic echocardiogram,^ *^SAVR^* ^surgical aortic valve replacement^
