# Supplementary material for: Decision making in treatment of symptomatic severe aortic stenosis: a survey study in Dutch heart centres
Source: Neth Heart J. 2022 Apr 5;30(9):423–8. doi: 10.1007/s12471-022-01676-w (PMC9402830; doi:10.1007/s12471-022-01676-w)
Supplement: Supplementary file 3 — Table S2. Guidelines and care-paths [file 12471_2022_1676_MOESM3_ESM.docx]

|  | **Academic hospitals (*n*=8)** | **Large teaching hospitals (*n*=8)** | **Total (*n*=16)** |
| --- | --- | --- | --- |
|  | ***n* (%)** | ***n* (%)** | ***n* (%)** |
| **Guidelines** |  |  |  |
| AHA/ACC | 2 (25) | 0 (0) | 2 (13) |
| ESC/EACTS | 7 (88) | 7 (88) | 14 (88) |
| Decision pathway | 1 (13) | 0 (0) | 1 (6) |
| Moments of decision NVT | 3 (38) | 2 (25) | 5 (31) |
| Indication document TAVR | 3 (38) | 5 (63) | 8 (50) |
| Local protocol | 1 (13) | 3 (38) | 4 (25) |
| Other: NVVC THI protocol | 1 (13) | 0 (0) | 1 (6) |
| **Care path** |  |  |  |
| *Reason for postponing treatment decision* |  |  |  |
| Consultation other than with cardiology |  |  |  |
| Seldom to never | 1 (13) | 0 (0) | 1 (6) |
| Sometimes to regularly | 7 (88) | 8 (100) | 15 (94) |
| Often to always | 0 (0) | 0 (0) | 0 (0) |
| Additional examinations |  |  |  |
| Seldom to never | 0 (0) | 0 (0) | 0 (0) |
| Sometimes to regularly | 7 (88) | 8 (100) | 15 (94) |
| Often to always | 1 (13) | 0 (0) | 1 (6) |
| Doubts about patient's vitality |  |  |  |
| Seldom to never | 1 (13) | 1 (13) | 2 (13) |
| sometimes to regularly | 7 (88) | 7 (88) | 14 (88) |
| Often to always | 0 (0) | 0 (0) | 0 (0) |
| Patient's symptoms not clear |  |  |  |
| Seldom to never | 4 (50) | 5 (63) | 9 (56) |
| Sometimes to regularly | 4 (50) | 3 (38) | 7 (44) |
| Often to always | 0 (0) | 0 (0) | 0 (0) |
| Presence of outpatient clinic with carousel approach |  |  |  |
| For TAVR | 1 (13) | 0 (0) | 1 (6) |
| For SAVR | 2 (25) | 0 (0) | 2 (13) |
| For both SAVR and TAVR | 4 (50) | 5 (63) | 9 (56) |
| None | 1 (13) | 3 (38) | 4 (25) |
| *Moment of preoperative assessment by* |  |  |  |
| Cardiothoracic surgeon |  |  |  |
| Before heart team meeting | 0 (0) | 0 (0) | 0 (0) |
| After heart team meeting without carousel | 2 (25) | 1 (13) | 3 (19) |
| After heart team in outpatient clinic with carousel | 5 (63) | 4 (50) | 9 (56) |
| Day before operation | 1 (13) | 2 (25) | 3 (19) |
| None | 0 (0) | 1 (13) | 1 (6) |
| Interventional cardiologist |  |  |  |
| Before heart team meeting | 1 (13) | 0 (0) | 1 (6) |
| After heart team meeting without carousel | 4 (50) | 4 (50) | 8 (50) |
| After heart team in outpatient clinic with carousel | 1 (13) | 3 (38) | 4 (25) |
| Day before operation | 2 (25) | 1 (13) | 3 (19) |
| None | 0 (0) | 0 (0) | 0 (0) |
| Nurse practitioner/physician assistant |  |  |  |
| Before heart team meeting | 0 (0) | 2 (25) | 2 (13) |
| After heart team meeting without carousel | 0 (0) | 2 (25) | 2 (13) |
| After heart team in outpatient clinic with carousel | 6 (75) | 3 (38) | 9 (56) |
| Day before operation | 0 (0) | 1 (13) | 1 (6) |
| None | 2 (25) | 0 (0) | 2 (13) |
| Nurse |  |  |  |
| Before heart team meeting | 0 (0) | 0 (0) | 0 (0) |
| After heart team meeting without carousel | 0 (0) | 0 (0) | 0 (0) |
| After heart team in outpatient clinic with carousel | 2 (25) | 0 (0) | 2 (13) |
| Day before operation | 4 (50) | 7 (88) | 11 (69) |
| None | 2 (25) | 1 (13) | 3 (19) |
| Clinical geriatrician |  |  |  |
| Before heart team meeting | 1 (13) | 1 (13) | 2 (13) |
| After heart team meeting without carousel | 3 (38) | 2 (25) | 5 (31) |
| After heart team in outpatient clinic with carousel | 2 (25) | 2 (25) | 4 (25) |
| Day before operation | 0 (0) | 0 (0) | 0 (0) |
| None | 2 (25) | 3 (38) | 5 (31) |
| Anaesthesiologist |  |  |  |
| Before heart team meeting | 0 (0) | 0 (0) | 0 (0) |
| After heart team meeting without carousel | 0 (0) | 0 (0) | 0 (0) |
| After heart team in outpatient clinic with carousel | 6 (75) | 5 (63) | 11 (69) |
| Day before operation | 2 (25) | 3 (38) | 5 (31) |
| None | 0 (0) | 0 (0) | 0 (0) |

**Table S2** Guidelines and care paths

*^AHA/ACC^* ^Guideline for the Management of Patients With Valvular Heart Disease [1],^*^, Decision Pathway^* ^Expert Consensus Decision Pathway for Transcatheter Aortic Valve Replacement in the Management of Adults With Aortic Stenosis [2],^ *^ESC/EACTS^* ^Guidelines for the management of valvular heart disease [3],^ *^Indicatie document THI^* ^Indication document Transcatheter Aortic Valve Intervention [Indicatiedocument Transcatheter Aortaklep Interventie] [4],^ *^Moments of Decision NVT^* ^Moments of decision making in the pre-, per- and postoperative trajectory of the cardiac surgical patient [Beslismomenten pre-, per- en postoperatieve traject van de hartchirurgische patiënt] [5],^ *^SAVR^* ^Surgical aortic valve replacement,^ *^TAVR^* ^Transcatheter aortic valve replacement^

1 Nishimura RA, Otto CM, Bonow RO, et al. 2014 AHA/ACC guideline for the management of patients with valvular heart disease: a report of the American College of Cardiology/American Heart Association Task Force on Practice Guidelines. J Thorac Cardiovasc Surg. 2014;148:e1-e132.

2 Otto CM, Kumbhani DJ, Alexander KP, et al. 2017 ACC Expert Consensus Decision Pathway for Transcatheter Aortic Valve Replacement in the Management of Adults With Aortic Stenosis: A Report of the American College of Cardiology Task Force on Clinical Expert Consensus Documents. J Am Coll Cardiol. 2017;69:1313-1346.

3 Baumgartner H, Falk V, Bax JJ, et al. 2017 ESC/EACTS Guidelines for the Management of Valvular Heart Disease. Rev Esp Cardiol (Engl Ed). 2018;71:110.

4 Nederlandse Vereniging voor Cardiologie, Nederlandse vereniging voor thoraxchirurgie. Indicatiedocument Transcatheter Aortaklep Interventie. 2017.

5 Nederlandse vereniging voor thoraxchirurgie, BolRaap G, Drijver YN, et al. Beslismomenten pre-, per en postoperatieve traject van de hartchirurgische patiënt. 2014.
