# Supplementary material for: Decision making in treatment of symptomatic severe aortic stenosis: a survey study in Dutch heart centres
Source: Neth Heart J. 2022 Apr 5;30(9):423–8. doi: 10.1007/s12471-022-01676-w (PMC9402830; doi:10.1007/s12471-022-01676-w)
Supplement: Supplementary file 4 — Table S3. Screening data in referring hospital, heart centre and heart team meeting [file 12471_2022_1676_MOESM4_ESM.docx]

|  | **Academic hospitals (*n*=8)** | **Large teaching hospitals (*n*=8)** | **Total (*n*=16)** |
| --- | --- | --- | --- |
|  | ***n* (%)** | ***n* (%)** | ***n* (%)** |
| Data requested from referring hospital |  |  |  |
| *Frailty*  FrailtyCardiothoracic surgeon |  |  |  |
| Never-seldom  Interventional cardiologist | 2 (25) | 4 (50) | 6 (38) |
| Sometimes-regularly | 5 (63) | 4 (50) | 9 (56) |
| Often-always | 1 (13) | 0 (0) | 1 (6) |
|  |  |  |  |
| *Cognition/mood* |  |  |  |
| Never-seldom  Interventional cardiologist | 2 (25) | 3 (38) | 5 (31) |
| Sometimes-regularly | 5 (63) | 5 (63) | 10 (63) |
| Often-always | 1 (13) | 0 (0) | 1 (6) |
|  |  |  |  |
| *Nutritional status* |  |  |  |
| Never-seldom  Interventional cardiologist | 5 (63) | 4 (50) | 9 (56) |
| Sometimes-regularly | 2 (25) | 4 (50) | 6 (38) |
| Often-always | 1 (13) | 0 (0) | 1 (6) |
|  |  |  |  |
| *Physical functioning/functionality in (I)ADL* |  |  |  |
| Never-seldom  Interventional cardiologist | 3 (38) | 2 (25) | 5 (31) |
| Sometimes-regularly | 4 (50) | 6 (75) | 10 (63) |
| Often-always | 1 (13) | 0 (0) | 1 (6) |
| Data obtained in heart centre |  |  |  |
| *Frailty*  FrailtyCardiothoracic surgeon |  |  |  |
| Never-seldom  Interventional cardiologist | 0 (0) | 1 (13) | 1 (6) |
| Sometimes-regularly | 3 (38) | 2 (25) | 5 (31) |
| Often-always | 5 (63) | 5 (63) | 10 (63) |
|  |  |  |  |
| *Cognition/mood* |  |  |  |
| Never-seldom  Interventional cardiologist | 0 (0) | 1 (13) | 1 (6) |
| Sometimes-regularly | 4 (50) | 2 (25) | 6 (38) |
| Often-always | 4 (50) | 5 (63) | 9 (56) |
|  |  |  |  |
| *Nutritional status* |  |  |  |
| Never-seldom  Interventional cardiologist | 0 (0) | 1 (13) | 1 (6) |
| Sometimes-regularly | 3 (38) | 2 (25) | 5 (31) |
| Often-always | 5 (63) | 5 (63) | 10 (63) |
|  |  |  |  |
| *Physical functioning/functionality in (I)ADL* |  |  |  |
| Never-seldom  Interventional cardiologist | 0 (0) | 1 (13) | 1 (6) |
| Sometimes-regularly | 4 (50) | 1 (13) | 5 (31) |
| Often-always | 4 (50) | 6 (75) | 10 (63) |
| Data available at heart team meeting |  |  |  |
| *Frailty*  FrailtyCardiothoracic surgeon |  |  |  |
| Never-seldom  Interventional cardiologist | 2 (25) | 3 (38) | 5 (31) |
| Sometimes-regularly | 6 (75) | 3 (38) | 5 (56) |
| Often-always | 0 (0) | 2 (25) | 2 (13) |
|  |  |  |  |
| *Cognition/mood* |  |  |  |
| Never-seldom  Interventional cardiologist | 1 (13) | 1 (13) | 2 (13)  ) |
| Sometimes-regularly | 7 (88) | 4 (50) | 11 (69) |
| Often-always | 0 (0) | 3 (38) | 3 (19) |
|  |  |  |  |
| *Nutritional status* |  |  |  |
| Never-seldom  Interventional cardiologist | 3 (38) | 3 (38) | 6 (38) |
| Sometimes-regularly | 4 (50) | 4 (50) | 8 (50) |
| Often-always | 1 (13) | 1 (13) | 2 (13) |
|  |  |  |  |
| *Physical functioning/functionality in (I)ADL* |  |  |  |
| Never-seldom  Interventional cardiologist | 1 (13) | 1 (13) | 2 (13) |
| Sometimes-regularly | 7 (88) | 3 (38) | 10 (63) |
| Often-always | 0 (0) | 4 (50) | 4 (25) |
|  |  |  |  |
|  |  |  |  |

**Table S3** Screening data in referring hospital, heart centre and heart team meeting

*ADL* activities of daily living, *IADL* instrumental activities of daily living
